# Exact spectral function of one-dimensional Bose gases

**DOI:** 10.1093/nsr/nwaf294

**Published:** 2025-07-21

**Authors:** Song Cheng, Yang-Yang Chen, Xi-Wen Guan, Wen-Li Yang, Rubem Mondaini, Hai-Qing Lin

**Affiliations:** Institute of Modern Physics, Northwest University, Xi’an 710069, China; Department of Physics, The University of Hong Kong, Hong Kong 999077, China; Beijing Computational Science Research Center, Beijing 100193, China; Institute of Modern Physics, Northwest University, Xi’an 710069, China; Shaanxi Key Laboratory for Theoretical Physics Frontiers, Xi’an 710069, China; Innovation Academy for Precision Measurement Science and Technology, Chinese Academy of Sciences, Wuhan 430071, China; Department of Fundamental and Theoretical Physics, Research School of Physics, Australian National University, Canberra ACT 0200, Australia; Peng Huanwu Center for Fundamental Theory, Xi’an 710069, China; Institute of Modern Physics, Northwest University, Xi’an 710069, China; Shaanxi Key Laboratory for Theoretical Physics Frontiers, Xi’an 710069, China; Peng Huanwu Center for Fundamental Theory, Xi’an 710069, China; Department of Physics, University of Houston, Houston, Texas 77004, USA; Institute for Advanced Study in Physics and School of Physics, Zhejiang University, Hangzhou 310058, China

**Keywords:** quantum integrable systems, low-dimensional quantum gas, spectral function, nonlinear Luttinger liquid

## Abstract

Exactly solved models provide rigorous understanding of many-body phenomena in strongly correlated systems. In this article, we report a breakthrough in uncovering universal many-body correlated properties of the quantum integrable Lieb–Liniger model. We exactly calculate the dynamical correlation functions by computing the form factors through a newly developed method, by which we are capable of calculating all possible ‘relative excitations’ over the ground state or a finite temperature state to high precision. Consequently, full spectral functions obtained for the model manifest the unique power-law singularity behaviour at the spectral threshold, confirming the validity of nonlinear Luttinger liquid theory. Our method advances the theory of dynamical correlation functions with high precision towards the thermodynamic limit, and is capable of benchmarking experimental observation of such novel correlated properties.

## Teaser

A powerful algorithm for rigorously calculating spectral function of the Lieb-Liniger model was established, essentially confirming universal edge singularity for the first time.

## INTRODUCTION

The novel phenomena associated with strongly correlated systems such as the Mott phase transition, spin-charge separation and Fermi edge singularity (FES) have fascinated condensed matter physicists [1,2]. A significant way to enhance the correlation for interacting quantum systems is to reduce the spatial dimensionality, which simplifies complicated situations in reality and thereby helps a lot in resolving the puzzles [3,4]. In this regard, the key to understanding the underlying physics of quantum many-body systems is to discover the behaviour of correlation functions. Nevertheless, from a theoretical perspective, despite very few limiting cases, such as the Tonks–Girardeau gas with various confinements [5–9], a rigorous calculation of correlation functions remains a formidable task, even on an account of the results obtained by quantum Monte Carlo and density matrix renormalization group methods. From an experimental perspective, the past few decades have witnessed successful developments in ultracold atoms with unprecedented levels of manipulation and control [10,11]. So far, only a few kinds of correlation functions in one-dimensional (1D) systems have been measured, such as the momentum distribution via optical imaging [12,13], the spectral function via photoemission spectroscopy [14] and momentum-resolved Raman spectroscopy [15], the two-point correlation function of a dynamical structure factor via Bragg scattering spectroscopy [16–19], etc. Such novel progress has therefore stimulated great demand for exact results on miscellaneous correlation functions in 1D systems; see also the recent observation of the dissipative dynamics in 1D interacting ultracold atoms [20].

Based on the Bethe ansatz [21,22], recent developments of the nonperturbative physics of exactly solved models either in or out of equilibrium are very stimulating [23–28]. However, despite decades of extensive research, a rigorous understanding and exact computation of dynamical correlation functions at a many-body level have remained elusive. In this paper, by extending the *form factor* to large-size systems and dividing the infinite dimensions of the Hilbert space into a series of subspaces for arbitrary ‘relative excitations’, a highly efficient algorithm is established for exactly calculating many-body correlated properties of quantum integrable systems. As an exotic application, we rigorously compute various correlation functions of 1D Bose gases, i.e. the Lieb–Liniger model, at unprecedented system sizes, giving exact results of the spectral function beyond the accuracy of so-far existing numerical calculations of correlation functions for this model. Our algorithm is powerful enough to successfully capture power-law singularities in the vicinities of spectral thresholds, and certainly shows different advantages from other methods, for example, the ABACUS algorithm developed by Caux and his collaborators [29–32]. Our results suggest that only a system size up to thousands of particles can guarantee enough resolution to reach the true power law of the correlations at the spectral edges, thus further confirming the validity of the nonlinear Tomonaga–Luttinger liquid [33–36], and ensuring rigorous access to the emergent behaviour of correlation functions [37] in the thermodynamic limit.

## MODEL

We now apply our algorithm to calculate the spectral function of the Lieb–Liniger model (LLM) [38] that describes *N* bosons confined on a line of length *L* with contact interaction. The exact solution of the model [21,39] benchmarks a large variety of many-body phenomena, ranging from universal thermodynamics and quantum criticality [39] to correlation functions [21,29–31,37,40–49] and the behaviour of the Tomonaga–Luttinger liquid (TLL) [33,35,36,50]. Such theoretical developments have inspired unprecedented levels of experimental study in ultracold atoms [13,17,19]; see [11].

The Hamiltonian of the LLM reads


(1) \begin{eqnarray*}
H = - \sum _{i=1}^{N} \frac{\partial ^2}{\partial x_i^2} + 2c \sum _{i>j}^{N} \delta ( x_i - x_j ),
\end{eqnarray*}

where $c>0$ ($c<0$) stands for repulsion (attraction), and a dimensionless parameter $\gamma =c L/N$ is introduced for the interaction strength [38]. Hereafter, only the repulsive interaction will be taken into account. With the help of the Bethe ansatz, solving the eigenvalue problem of Hamiltonian (1) reduces to solving the transcendental Bethe ansatz equations


(2) \begin{eqnarray*}
\lambda _j + \frac{1}{L} \sum _{k=1}^{N} \theta ( \lambda _j - \lambda _k ) = \frac{2\pi }{L} I_j, \quad j = 1, \dots , N,
\end{eqnarray*}

where $\theta (x)=2\arctan (x/c)$, pseudomomenta $\lbrace \lambda _j\rbrace$ are distinct real numbers and quantum numbers (QNs) $\lbrace I_j\rbrace$ are distinct integers (half-integers) if *N* is odd (even). There is a one-to-one map between a set of QNs and a set of pseudomomenta, utilizing which leads to expressions for the total momentum and energy of the system: $P_{\lbrace \lambda \rbrace }=\sum _{j=1}^{N} \lambda _j, \, E_{\lbrace \lambda \rbrace }=\sum _{j=1}^{N} \lambda _j^2$. The ground state is formulated by a Fermi-sea-like distribution for QNs (i.e. $I=\lbrace -{(N-1)}/{2},\dots , {(N-1)}/{2} \rbrace$). In this model, $P_\mathrm{m}$, one of the tag quantum numbers, connects the excited momentum through $P_\mathrm{m}=PL/2\pi$.

The spectral function (SF) in general represents the probability of tunneling a particle with specified momentum and energy into or out of the system. Let us start from the single-particle Green function


(3) \begin{eqnarray*}
{i}\cdot \mathcal {G}(x,t) \equiv \langle \mathcal {T} [ \Psi (x,t)\Psi ^\dagger (0,0) ] \rangle _N,
\end{eqnarray*}

where $\langle \cdot \rangle _N$ means that the expectation value is taken over the ground state of an *N*-particle system, and $\Psi (x,t)$ is the bosonic field operator. For simplicity, we merely consider the larger Green function $G^>(x,t)$, with the treatment for the lesser one similar. Inserting a completeness relation into the two field operators yields


(4) \begin{eqnarray*}
{i}&&\cdot G^> (x,t)= \sum _{\lbrace \mu \rbrace _{N+1}} \frac{ \langle \lbrace \lambda \rbrace _{N} | \Psi (x,t) | \lbrace \mu \rbrace _{N+1}\rangle \, \langle \lbrace \mu \rbrace _{N+1} | \Psi ^\dagger (0,0) | \lbrace \lambda \rbrace _N\rangle }{ \langle \lbrace \lambda \rbrace _N| \lbrace \lambda \rbrace _N\rangle \, \langle \lbrace \mu \rbrace _{N+1}| \lbrace \mu \rbrace _{N+1}\rangle },
\end{eqnarray*}

where $| \lbrace \nu \rbrace _M \rangle$ is an eigenstate consisting of *M* particles and specified by a set of pseudomomenta $\lbrace \nu \rbrace _M$. The ground state and intermediate state are respectively denoted by $| \lbrace \lambda \rbrace _N \rangle$ and $| \lbrace \mu \rbrace _{N+1} \rangle$. The *form factor* of the field operator is $\mathcal {F}(\lbrace \lambda \rbrace _N,\lbrace \mu \rbrace _{N+1}) = \langle \lbrace \mu \rbrace _{N+1} | \Psi ^\dagger (0,0) | \lbrace \lambda \rbrace _N\rangle$. Based on this notation, we have


(5) \begin{eqnarray*}
&& {i} \cdot G^>(x,t)\\
&&= \sum _{\lbrace \mu \rbrace _{N+1}} e^{{i} \phi ^+ } \frac{|\mathcal {F}(\lbrace \lambda \rbrace _N,\lbrace \mu \rbrace _{N+1})|^2}{\Vert \lbrace \mu \rbrace _{N+1} \Vert ^2 \cdot \Vert \lbrace \lambda \rbrace _N \Vert ^2}
\end{eqnarray*}

with $\phi ^+= (E_{\lbrace \lambda \rbrace }-E_{\lbrace \mu \rbrace })t-(P_{\lbrace \lambda \rbrace }-P_{\lbrace \mu \rbrace })x$. According to the definition of the SF $\mathcal {A}(k,\omega ) = -({1}/{\pi } ) {Im}\, \mathcal {G}(k,\omega )$, where $\mathcal {G}(k,\omega )$ is the Fourier transform of $\mathcal {G}(x,t)$, one finally obtains


(6) \begin{eqnarray*}
&&{\frac{\mathcal {A}(k,\omega )}{L}}\\ &&= \sum _{\lbrace \mu \rbrace _{N+1}} \frac{ \delta _{k,P_{ \lbrace \mu \rbrace ,\lbrace \lambda \rbrace }} \delta (\omega - E_{\lbrace \mu \rbrace ,\lbrace \lambda \rbrace } ) |\mathcal {F}(\lbrace \lambda \rbrace _N,\lbrace \mu \rbrace _{N+1})|^2}{\Vert \lbrace \mu \rbrace _{N+1} \Vert ^2 \cdot \Vert \lbrace \lambda \rbrace _N \Vert ^2}\\
&& + \sum _{\lbrace \mu \rbrace _{N-1}} \frac{ \delta _{-k,P_{ \lbrace \mu \rbrace ,\lbrace \lambda \rbrace }} \delta (\omega + E_{\lbrace \mu \rbrace ,\lbrace \lambda \rbrace } ) |\mathcal {F}(\lbrace \mu \rbrace _{N-1},\lbrace \lambda \rbrace _{N})|^2}{\Vert \lbrace \mu \rbrace _{N-1} \Vert ^2 \cdot \Vert \lbrace \lambda \rbrace _N \Vert ^2},\!\!\!\!\!\!\! \\
\end{eqnarray*}

where $\delta _{n,m}$ is the Kronecker delta function and $C_{\lbrace \mu \rbrace ,\lbrace \lambda \rbrace }\equiv C_{\lbrace \mu \rbrace }-C_{\lbrace \lambda \rbrace }$ if $C=P$ or *E*. It is notable that the SF we adopt here leads to the summation of Fourier transforms of the greater and lesser Green functions [9], instead of their difference. This customary definition is introduced because the conventional SF may result in negative spectral weights when $\omega <0$ for a bosonic system. In addition, for the sake of clarity, we hereafter focus on the SF $A(k,\omega ) = \mathcal {A} (k,\omega -h)$, where *h* is the chemical potential. The validity of the result is quantitatively checked by the saturation of the sum rule


(7) \begin{eqnarray*}
\sum _{k} \int _{-\infty }^{0} \frac{ {d}\omega }{ N} A(k,\omega ) = 1.
\end{eqnarray*}

## ALGORITHM

Given an operator $\hat{O}$, its *form factor* is defined by $\mathcal {F} (|r\rangle ,|s\rangle ) = \langle s | \hat{O} |r \rangle$, i.e. the matrix element evaluated between two eigenstates of the interacting Hamiltonian [21]. The time-dependent correlation function is expressed as the spectral representation


(8) \begin{eqnarray*}
&&\langle s| \hat{O}^\dagger (x,t) \hat{O}(0,0) | s \rangle \\
&&= \sum _{|r\rangle \in \mathfrak {E}} e^{\mathrm{i} (E_{r,s}t - P_{r,s}x)} \frac{|\mathcal {F}(|s\rangle ,|r\rangle ) |^2}{ \Vert s \Vert ^2 \, \Vert r \Vert ^2},
\end{eqnarray*}

where $|s\rangle$ is the eigenstate under study, $\mathfrak {E}$ is the eigenspace of the Hamiltonian, $E_{r,s}=E_r-E_s$ ($P_{r,s}=P_r -P_s$) stands for the difference in energy (momentum) between $|r\rangle$ and $|s\rangle$, and $\Vert \cdot \Vert$ is the norm of a state. State $|s\rangle$ is not limited to the ground state; however, in some circumstances, such as finite temperature and post-quench, it may be a highly excited state.

Note that the summation is assumed to include all the eigenstates, and the evaluation of Equation (8) is nothing but counting the elements of $\mathfrak {E}$ together with calculating their form factors. It is obvious that the key step is to efficiently and quickly find the essential states in the process of navigating $\mathfrak {E}$. To this end, we have developed an algorithm suitable for calculating various dynamical correlation functions, such as the dynamical structure factor in the ground state [37] and the one-body dynamical correlation at finite temperatures [44]. The seminal idea is that the most relevant states for calculating form factors of a local observable ought not be much different from the state of our interest in the perspective of the QN configuration. In light of the prospectively unified description for any state under investigation, called the *reference state* below, one has to abandon the conventional understanding of particle-hole pairs of excitation over the Fermi sea. We introduce the concept of ‘relative excitation’ that leads to the re-distribution of QNs away from the reference state; see the online supplementary material for an explicit example.

For the purpose of classifying the excited states over a reference state, we introduce a set of four tags ($P_m, N_p, P_l, N_l$). They are four non-negative integers, with the wave number $P_m = k L/2\pi$ specifying the excited momentum, $N_p$ the number of particles involved in the ‘relative excitation’, $N_l < N_p$ the number of particles jumping leftward and $P_l \ge N_l$ the sum of excited momentum due to those $N_l$ leftward particles in units of $2\pi /L$. They can be seen as a set of QNs to describe the ‘relative excitation’ over a reference state. In this way, $\mathfrak {E}$ is separated into a large number of subspaces, and one may find the most relevant states through a proper choice of tags. Since this partition for $\mathfrak {E}$ dynamically depends on the reference state, our algorithm is very efficient in accelerating the search of those relevant states that make non-negligible contributions; see the example table within the online supplementary material. Consequently, this method is especially efficient for tackling highly excited states, for instance at finite temperature or the post-quench steady state. Moreover, our algorithm considers momentum as the first quantum number, which makes it convenient to access the line shape of dynamical correlations as well as to directly benchmark the experimental data. All these features, distinct from the ABACUS algorithm [32], demonstrate the advantage of our algorithm to uncover the power-law behaviour of the spectral function at the spectral threshold.

## RESULTS

**Figure 1.**
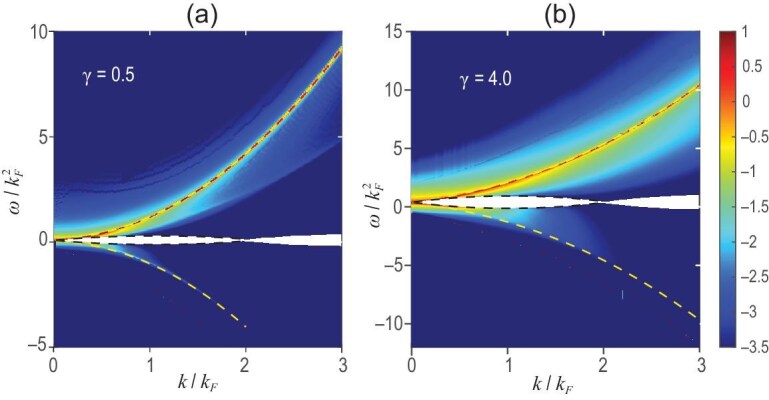
The momentum-energy-resolved SF of the Lieb–Liniger gas in the ground state of system size $N=L=100$. Panels (a) and (b) respectively show interaction strengths $\gamma =0.5$ and 4.0 with sum rules 0.9999 and 0.9935. Momentum and energy are respectively given in units of the Fermi energy and Fermi momentum, defined by $k_F = \pi N/L$. For the sake of clarity, we adopt the logarithm of the SF, such that the higher the value, the brighter the color. The yellow (black) dashed line is the type-I (type-II) dispersion relation.

**Figure 2.**
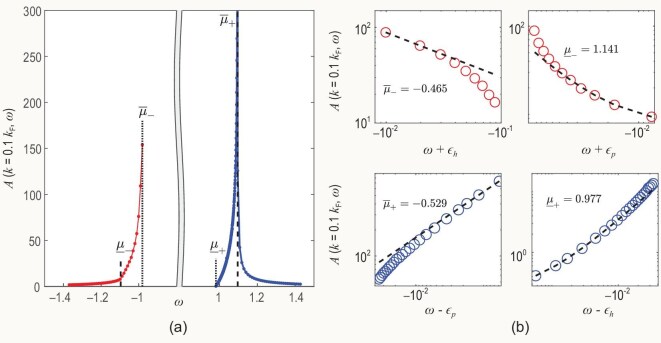
SF versus energy for momentum $k=0.1k_F$ and interaction strength $\gamma =4.0$. The system size is $N=L=4000$, and the energy is measured in units of the Fermi energy. (a) The full power-law feature of the SF with varying energy, where the red (blue) curve represents the emission (absorption) spectrum, and the black dashed (dotted) lines represent the thresholds of the single-particle spectrum $\pm \epsilon _p$ ($\pm \epsilon _h$). Apparently, there exists a peak around the type-I (type-II) dispersion in the absorption (emission) spectrum. The blank region between two doted-lines is also indicated between the two type-II excitations in Fig. 1. (b) The singularity powers of the SF in the vicinities of the thresholds of the single-particle spectrum. A log-log coordinate is used for the sake of clarity. The red (blue) circles represent the SF in the emission (absorption) spectrum and the black dashed lines represent power-law curves predicted by Equation (9), where the corresponding exponents $\overline{\mu }_-$, $\underline{\mu }_-$, $\overline{\mu }_+$ and $\underline{\mu }_+$ are $-0.465$, 1.141, $-0.529$ and 0.977, in good agreement with the nonlinear TLL predictions $-0.422$, 0.934, $-0.501$, and 1.043, respectively.

The logarithm of the SF for the LLM is shown in Fig. 1 with system size $N=L=100$. Here the focus is on the intermediate interaction and weak interaction situations, i.e. $\gamma =4.0$ and $\gamma =0.5$, respectively. The yellow (black) dashed lines represent type-I (type-II) dispersion relations, corresponding to the creation of a particle (hole) outside (inside) of the Fermi sea [21,38]. Single-particle and single-hole excitations defining the edges of spectra feature the nontrivial role of interaction in 1D many-body systems. Comparing panels (a) and (b) of Fig. 1, we see that the energy of the hole excitation is suppressed when the interaction strength $\gamma$ decreases, but that the type-II dispersion exists as long as $\gamma$ does not vanish. This phenomenon underlies why the Bogoliubov approximation is deficient, which was first discovered through the Bethe ansatz solution for this model [38]. It is obvious that either the absorption or emission spectrum is separated into three regions, all of which are determined by pairs of p-h excitations. Let us denote the type-I (type-II) dispersion as $\epsilon _p$ ($\epsilon _h$) in the particle sector $\omega >0$, and as $-\epsilon _p$ ($-\epsilon _h$) in the hole sector $\omega <0$. For the half plane of $\omega >0$, below $\epsilon _h$ is blank, implying that no excitation bears that energy and momentum, which is also clearly seen in Fig. 2. For $\epsilon _h < \omega < \epsilon _p$, a spectral continuum consisting of states produced by arbitrary pairs of p-h excitations occurs, while for $\omega >\epsilon _p$, it is the region where the excitations involve the states generated by two, three and multiple p-h pairs.

In Fig. 2(a), we show the line shape of the SF versus energy with fixed momentum $k=0.1k_F$ and interaction strength $\gamma =4.0$. It should be noted that the system size here is $N=L=4000$, different from Fig. 1. The particle-hole asymmetry is evidenced by the blue (red) curve within the absorption (emission) spectrum exhibiting a peak around the type-II (type-I) dispersion. Their birth mechanisms are distinct: the red peak mainly comes from the single p-h excitation, while the blue peak mainly comes from the situation of multiple p-h pairs.

In Fig. 2(b) the fascinating many-body FES phenomenon is clearly observed, which is a typical impurity problem closely related to orthogonal catastrophe [1,4]. The FES manifests as the SF on the thresholds of spectra displaying power-law behaviour [33,35,36]:


(9) \begin{eqnarray*}
A(k,\omega ) \sim {const} + | \omega \mp \epsilon _{p,h} | ^{\mu _\pm }
\end{eqnarray*}

with subscript $+$ ($-$) specifying the edge exponent on the absorption (emission) threshold. The original FES arises from the transient potential brought forth by a deep electron excitation, which leaves behind a core hole and scatters with the noninteracting electrons in the conduction band [1]. It was interpreted as an impurity problem, i.e. an impurity moving in a Fermi liquid. By the inclusion of interactions between particles, it reformed into a Luttinger liquid instead [2]. However, the conventional TLL only identifies the power law, and its particle-hole symmetry at the long-wavelength limit prevents it from distinguishing four thresholds [2,4]. This ambiguity disappears if we take into account the nonlinearity of the spectrum by combining bosonization and quantum integrable theory [33]. In our method, it is obvious that the threshold behaviour of the SF in the thermodynamic limit naturally emerges only when the energetic resolution narrows down to and is even lower than $10^{-2} k_F^2$, which in turn requires a very large system size of $N=L=4000$. Using the log-log coordinates, the exponents represented by dashed lines are readily obtained: $\overline{\mu }_-$, $\underline{\mu }_-$, $\overline{\mu }_+$ and $\underline{\mu }_+$ are $-0.465$, 1.141, $-0.529$ and 0.977. Our results confirm for the first time the validity of the power-law behaviour predicted from the nonlinear TLL with exponents $-0.422$, 0.934, $-0.501$ and 1.043 [33]. This reveals that the nonlinear TLL only validates in such a tiny region. In contrast, our algorithm is highly capable of evaluating the dynamical correlations with high precision in the whole energy region.

**Figure 3.**
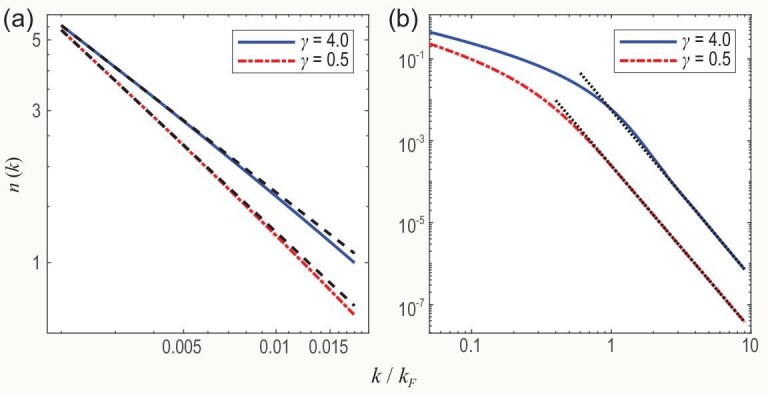
(a) The MDF in the TLL region. The black dashed lines represent the asymptotic powers $n(k) \sim k^\alpha$ with $\alpha =-0.750$ and $-0.908$ for $\gamma =4.0$ and 0.5, respectively. Here the momentum is very small, $k \rightarrow 0$. The numerical result of the powers further confirm the TLL predictions $\alpha =0.737$ and 0.894. Here we set $N=L=1000$ for our numerical calculation. (b) The MDF in the large momentum regime. The gradients of the black dotted lines show the asymptotic powers for a large momentum, $\alpha =-4.073$ and $-4.001$ for $\gamma =4.0$ and 0.5, respectively. They agree well with the power law for the large momentum tail $k^{-4}$. Tan’s contact is extracted as well: ${C}=0.595$ and 0.024 for $\gamma =4.0$ and 0.5, respectively. These values are in good agreement with the theoretical predictions 0.524 and 0.024. Here the system size is set as $N=L=100$.

Moreover, in Fig. 3, we study the power-law behaviour of the momentum distribution function (MDF), i.e. the static correlation $n(k) =\int _{-\infty }^{0} { \text{d}\omega } A(k,\omega )/{2\pi L}$ in the small and large momentum limits. The tail of the MDF for a system of contact interactions fulfills the universal law: Tan’s relation $\lim _{k \rightarrow \infty } n(k) = \mathcal {C} k^{-4}$. Here the weight $\mathcal {C}$ is Tan’s contact that builds wide relations with quantities such as the internal energy, pair correlation function and pressure. This asymptotic power-law behaviour is shown in Fig. 3(b), with the same system size $N=L=100$ as in Fig. 1. The exponents are extracted via the black dotted lines, with gradients $-4.00$ and $-4.07$ for $\gamma =0.5$ and 4.0, respectively. Tan’s contact $\mathcal {C}$ is theoretically calculated through thermodynamic Bethe ansatz equations, giving $\mathcal {C}=0.524$ and 0.024 for $\gamma =4.0$ and 0.5, respectively, in accordance with 0.595 and 0.024 obtained from Fig. 3(b). For the small momentum region shown in Fig. 3(a), it is well known that the MDF obeys an asymptotic power law $\lim _{k \rightarrow 0} n(k) \sim k^{{1}/{2K}-1}$ according to TLL theory [4], and an easy calculation gives the powers $-0.737$ and $-0.894$. Since the data of Fig. 1 are not sufficient for a visible resolution in momentum, we therefore make use of a larger system $N=L=1000$. The gradients of the black dashed lines are $-0.750$ and $-0.908$ for $\gamma =4.0$ and $\gamma =0.5$, respectively. Here we show that the results of different methods agree well with each other. This again indicates the high capability of our method to rigorously study the emergent behaviour of correlation functions appearing in the thermodynamic limit. A wide application of our finding to other integrable systems within the form-factor formalism is straightforward.

## CONCLUSIONS

The evaluation of correlation functions for a strongly correlated system in general is challenging. In this scenario, the quantum integrable models are of significant importance to benchmark observations in ultracold atomic experiments and other solid state materials. In this paper, building on the *form factors* and Bethe ansatz solution, we have presented an efficient algorithm to rigorously calculate the correlated properties of quantum integrable systems. In particular, we have obtained the spectral function of Lieb–Liniger Bose gases of arbitrary interaction strength with so far arguably the best precision. The spectral distribution on the full momentum-energy plane, the line shapes and especially the power-law behaviour of the dynamic correlations on spectral thresholds are explicitly presented. The power law of the static correlation function, i.e. the MDF, in the large and small momentum regions is obtained, as well as Tan’s contact $\mathcal {C}$, showing excellent agreement with the theoretical predictions.

It is worth emphasizing that the exponents describing Fermi edge singularities have been given explicitly for the system with the largest accessible size $N=L=4000$, which essentially confirms the nonlinear TLL. We have observed that such a huge system is indeed capable of necessitating the power law of correlation functions, so far beyond the capability of other methods. Hundreds of particles cannot guarantee the validation of such a power law.

Our algorithm efficiently deals with Fermi-sea-like quantum numbers, and is widely applicable to other quantum integrable systems in the presence of more intricate mathematical structures, that is, those that may possess string bound states, such as the attractive LLM, isotropic and anisotropic Heisenberg models, and those of multiple types of degrees of freedom, for instance the 1D SU(2) Fermi gases. Moreover, in combination with the local density approximation, it is well suited for systems with confinements, such as Bose gases in harmonic and linear traps.

In general, our work provides a rigorous approach to various emergent features of correlation functions for 1D strongly correlated systems either in or out of equilibrium such as the spectral function, the dynamical structure factor, quench dynamics, etc. This also opens new avenues for experimental verification in quantum gases and condensed matter systems.

## Supplementary Material

nwaf294_Supplemental_File
