# Supplementary material for: Exact spectral function of one-dimensional Bose gases
Source: Natl Sci Rev. 2025 Jul 21;12(9):nwaf294. doi: 10.1093/nsr/nwaf294 (PMC12404033; doi:10.1093/nsr/nwaf294)
Supplement: nwaf294_Supplemental_File [file nwaf294_supplemental_file.pdf]

Supplementary Material for The Exact Spectral Function of One-Dimensional Bose Gases

Song Cheng, Yang-Yang Chen, Xi-Wen Guan, Wen-Li Yang, Rubem Mondaini, and Hai-Qing Lin

THE DETERMINANT REPRESENTATIONS OF FORM FACTORS

We list the main results of form factors present in the article, and recommend Refs. [1–4] for those who are interested in derivations in detail. In particular, Ref. [1] offers a pedagogical access to the algebraci Bethe ansatz technique and the various methods in calculating correlated properties of quantum integrable models.

If $\{k\}_M$ satisfies the BA equations, then the norm square of eigenvector $|\{k\}_M\rangle$ is expressed by

$$\|\{k\}_M\|^2 \equiv \langle\{k\}_M|\{k\}_M\rangle = c^M \prod_{j>l\geq 1}^{M} \frac{k_{jl}^2 + c^2}{k_{jl}^2} \det{}_M \mathfrak{G}(\{k\}_M) \tag{1}$$

where $\mathfrak{G}(\{k\}_M)$ is the Gaudin matrix with entry

$$\mathfrak{G}_{jl}(\{k\}_M) = \delta_{jl}\left[L + \sum_{s=1}^{M} K(k_j, k_s)\right] - K(k_j, k_l) \tag{2}$$

and kernel function

$$K(x, y) = \frac{2c}{(x-y)^2 + c^2}. \tag{3}$$

The norm square for $\mathcal{F}(\{\lambda\}_N, \{\mu\}_{N+1})$ is

$$\|\mathcal{F}(\{\lambda\}_N, \{\mu\}_{N+1})\|^2 = c^{2N+1} \frac{\prod_{j>k\geq 1}^{N+1}(\mu_{jk}^2 + c^2)^2}{\prod_{a=1}^{N+1}\prod_{b=1}^{N}(\mu_a - \lambda_b)^2} \left(\det{}_N U(\{\lambda\}_N, \{\mu\}_{N+1})\right)^2, \tag{4}$$

where the $N \times N$ matrix $U(\{\lambda\}_N, \{\mu\}_{N+1})$ is a function of two sets of pseudomomenta

$$U_{jk}(\{\lambda\}_N, \{\mu\}_{N+1}) = \delta_{jk} \cdot \left(V_j^+ - V_j^-\right)/\mathrm{i} + \frac{\prod_{a=1}^{N}(\lambda_a - \mu_j)}{\prod_{a\neq j}^{N+1}(\mu_a - \mu_j)}\left(K(\mu_j - \mu_k) - K(\mu_N - \mu_k)\right), \tag{5}$$

$$V_j^\pm = \frac{\prod_{a=1}^{N}(\lambda_a - \mu_j \pm \mathrm{i}c)}{\prod_{a=1}^{N+1}(\mu_a - \mu_j \pm \mathrm{i}c)}. \tag{6}$$

EXPONENTS OF EDGE SINGULARITY

The exact exponents of edge singularity for spectral function of Lieb-Liniger model was firstly calculated by a combination of nonlinear TLL theory and quantum integrability [5]. In order to obtain the exponents, one needs to solve two integral equations. One is for the shift function [1]

$$F_B(\nu|\lambda) = \frac{\pi + \theta(\nu - \lambda)}{2\pi} + \frac{1}{2\pi}\int_{-q}^{q} \mathrm{d}\mu\, K(\nu, \mu) F_B(\mu|\lambda) \tag{7}$$

where $\theta(x) = 2\arctan(x/c)$ and $K(x, y)$ is defined by Eq. (3). Note that $q > 0$ is the cut-off of pseudomomentum for ground state in thermodynamic limit. The other one is for the change of total momentum when adding a particle (hole) with pseudomomentum $\lambda > q$ ($|\lambda| < q$) to the ground state. This change $k(\lambda)$ is expressed by

$$k(\lambda) = \pm\left(\lambda - \pi n + \int_{-q}^{q} \mathrm{d}\nu\, \theta(\lambda - \nu)\rho(\nu)\right) \tag{8}$$

where $\pm$ is to specify adding a particle or hole and $\rho(x)$ is the distribution of pseudomomenta in thermodynamic limit, governed by following integral equation

$$\rho(\lambda) = \frac{1}{2\pi} + \frac{1}{2\pi}\int_{-q}^{q} \mathrm{d}\mu K(\lambda,\mu)\rho(\mu). \tag{9}$$

These exponents therefore read

$$\overline{\mu}_{\pm} = \frac{1}{2}\left(\frac{\delta_+ - \delta_-}{2\pi}\right)^2 + \frac{1}{2}\left(\frac{\delta_+ + \delta_-}{2\pi}\right)^2 - 1, \tag{10}$$

$$\underline{\mu}_{\pm} = \frac{1}{2}\left(\frac{2}{\sqrt{K}} + \frac{\delta_+ - \delta_-}{2\pi}\right)^2 + \frac{1}{2}\left(\frac{\delta_+ + \delta_-}{2\pi}\right)^2 - 1 \tag{11}$$

where $\delta_{\pm} = 2\pi F_B(\pm q, \lambda)$ and K is the Luttinger parameter.

By taking $\overline{\mu}_+$ ($\underline{\mu}_+$) as an example, we outline the numerical treatment for above integral equations. As $\overline{\mu}_+$ ($\underline{\mu}_+$) lies on the threshold generated by adding 1-particle (2-particles close to the same Fermi point and 1-hole) to the ground state, the sign in Eq. (8) is positive (negative). One should solve the corresponding λ by using Eq. (8), and then substitute this λ into the shift function $F_B(\pm q|\lambda)$. With the help of Eq. (7), $\delta_{\pm}(k)$ and thus the exponent $\overline{\mu}_+$ ($\underline{\mu}_+$) is obtained.

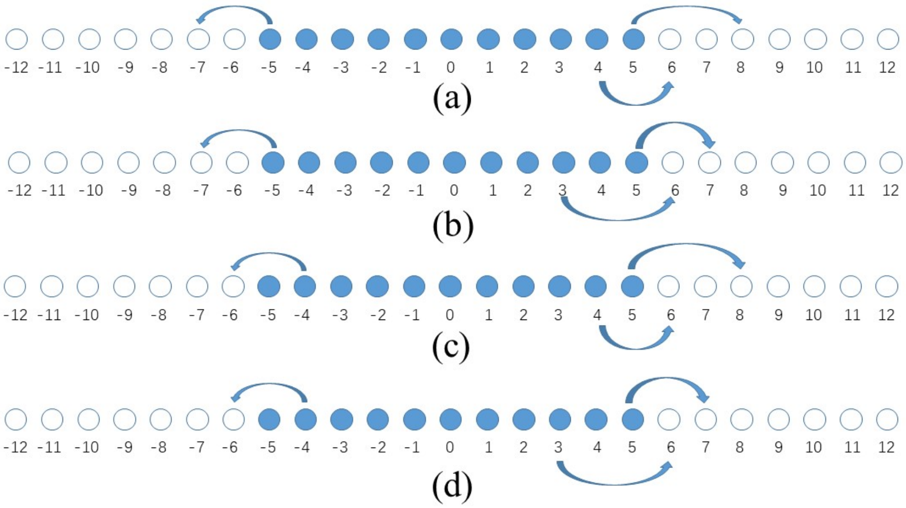

FIG. 1. A sketch to display the excited states sharing the same tag $P_\mathrm{m} = 3$, $N_\mathrm{p} = 3$, $P_\mathrm{l} = 2$, $N_\mathrm{l} = 1$. The system size is $N = 11$. We use balls and circles to stand for particles and holes respectively, and arrows to explain the movement of particles. The excitation generates states listed as below, (a): $\{-7,-4,-3,-2,-1,0,1,2,3,6,8\}$, (b): $\{-7,-4,-3,-2,-1,0,1,2,4,6,7\}$, (c): $\{-6,-5,-3,-2,-1,0,1,2,3,6,8\}$, (d): $\{-6,-5,-3,-2,-1,0,1,2,4,6,7\}$.

AN EXAMPLE: HOW TO COUNT THE STATES

Here we give an explicit example to show how the algorithm works.

At first, we show the way to produce excited states sharing the same tag in Figure 1. The system consists of $N = 11$ particles and we set $N_\mathrm{p} = 3$, $P_\mathrm{m} = 3$, $P_\mathrm{l} = 2$, $N_\mathrm{l} = 1$, without losing generality. As is described in the main text, the ground state is a line of continuous integers from -5 to 5. This tag set means 3 particles jumping outside of Fermi sea, 2 rightward and 1 leftward. The total shift of quantum numbers is $P_\mathrm{m} = 3$, and $P_\mathrm{r} = 5$ & $P_\mathrm{l} = 2$. The integers are the available quantum numbers (QNs), and the blue ball (circle) represents the corresponding QN is occupied (vacant) alias a particle (hole). The arrows is applied to specify the movement of particles in this excitation. There

Tags				Sum Rule			
P_m	N_p	P_l	N_l	N_l-sum rule	P_l-sum rule	N_p-sum rule	P_m-sum rule
10	1	0	0	0.0101	0.0101	0.0101	0.0140
	2	0	0	2.1387×10^{-4}	2.1387×10^{-4}	0.0038	
		1	1	0.0013	0.0013		
		2	1	6.7836×10^{-4}	6.7836×10^{-4}		
		3	1	4.3148×10^{-4}	4.3148×10^{-4}		
		4	1	3.0257×10^{-4}	3.0257×10^{-4}		
		5	1	2.2441×10^{-4}	2.2441×10^{-4}		
		6	1	1.7271×10^{-4}	1.7271×10^{-4}		
		7	1	1.3648×10^{-4}	1.3648×10^{-4}		
		8	1	1.1002×10^{-4}	1.1002×10^{-4}		
		9	1	9.0073×10^{-5}	9.0073×10^{-5}		
		10	1	7.4669×10^{-5}	7.4669×10^{-5}		
		⋮	⋮	⋮	⋮		
	3	0	0	4.4310×10^{-10}	4.4310×10^{-10}	1.1479×10^{-4}	
		1	1	3.2147×10^{-5}	3.2147×10^{-5}		
		2	1	1.8195×10^{-5}	1.8195×10^{-5}		
			1	N/A	N/A		
		4	1	9.6819×10^{-6}	1.0361×10^{-5}		
			2	6.7925×10^{-7}			
		5	1	7.7140×10^{-6}	8.9681×10^{-6}		
			2	1.2541×10^{-6}			
		6	1	6.3243×10^{-6}	7.9483×10^{-6}		
			2	1.6240×10^{-6}			
		7	1	5.2867×10^{-6}	7.1125×10^{-6}		
			2	1.8258×10^{-6}			
		8	1	4.4816×10^{-6}	6.3910×10^{-6}		
			2	1.9094×10^{-6}			
		9	1	3.8394×10^{-6}	5.7539×10^{-6}		
			2	1.9145×10^{-6}			
		10	1	3.3162×10^{-6}	5.1856×10^{-6}		
			2	1.8695×10^{-6}			
		⋮	⋮	⋮	⋮		

TABLE I. An example for the hole sector of spectral function with given excited momentum $k = 0.2k_F$ of system size $N = L = 100$ and interaction strength $\gamma = 4$. The X-sum rule is the total spectral weights of the states under tag X, such as N_p-sum rule specifying the contribution of different pairs of p-h excitation. Here N/A means there is no state under that tag.

is one particle jumping leftward out of Fermi sea with step length $P_\mathrm{l} = 2$. It is obvious that there are merely two possible arrangements for this leftward excitation, realized by moving either of the two neighbors (QNs -5 or -4) of left Fermi point. For the rightward excitation, the situation is a little bit different, the simultaneous movement of two QNs with total shift $P_\mathrm{r} = 5$. The step length of each movement should be 2 or 3, and the case of 4 and 1 is ruled out because it will generate repeated states belonging to other tags.

Following the production of excited states, Table I displays the data of one-body dynamical correlation function (the hole sector of spectral function $\omega < 0$) at a given excited momentum. The system size is $N = L = 100$, interaction strength is $\gamma = 4$, and the excited momentum is $k = 0.2k_F$. According to the introduction of algorithm in main text, $P_\mathrm{m} = 10$, $N_\mathrm{p} = 1, 2, 3$, and $P_\mathrm{l} = 0, 1, 2, \ldots, 10$. Note that N/A is exactly the case discussed before that repeated states belonging to other tags. Obviously the results show both N_p and P_l serve as good criteria for cut-off.

[1] V. E. Korepin, N. M. Bogoliubov, and A. G. Izergin, *Quantum Inverse Scattering Method and Correlation Functions* (Cambridge University Press, Cambridge, 1993).
[2] T. Kojima, V. E. Korepin, and N. A. Slavnov, Commun. Math. Phys. **188**, 657 (1997).
[3] T. Kojima, V. E. Korepin, and N. A. Slavnov, Commun. Math. Phys. **189**, 709 (1997).
[4] Jean-Sébastien Caux, and Pasquale Calabrese, and Nikita A. Slavnov, J. Stat. Mech. (2007) P01008.
[5] Adilet Imambekov, and Leonid I. Glazman, Phys. Rev. Lett. **100**, 206805 (2008).
